# Local investigation into the role of *Culicoides* species diversity (Diptera: Ceratopogonidae) in recurrent horse dermatitis cases in southwest France

**DOI:** 10.1186/s13071-025-06694-2

**Published:** 2025-03-05

**Authors:** Jorian Prudhomme, Clara Bardet, Ignace Rakotoarivony, Claire Garros, Émilie Bouhsira, Emmanuel Lienard

**Affiliations:** 1 INTHERES, Université de Toulouse, INRAE, ENVT, Toulouse, France; 2 Faculté de Pharmacie, UR EpidémioSurveillance and Circulation de Parasites Dans Les Environnements (ESCAPE), and ANSES, Université de Reims Champagne-Ardenne, USC Pathogènes-Environnement-Toxoplasme-Arthropodes-Réservoirs-bioDiversité (PETARD), Reims, France; 3 CIRAD, UMR ASTRE, 34398 Montpellier, France; 4 ASTRE, Univ Montpellier, CIRAD, INRAE, Montpellier, France

**Keywords:** Insect bite hypersensitivity, *Culicoides*, France

## Abstract

**Background:**

Insect bite hypersensitivity in horses (“sweet itch”) is a common pruritic, chronic, seasonal, and recurrent dermatitis affecting approximately 10% of horses in France and is a major concern for the horse industry and private owners. This dermatitis results from an allergic reaction to the saliva of specific biting flies (Diptera: Nematocera), primarily from the *Culicoides* genus. Given the frequent occurrence of this health problem and the limited investigation in France, we conducted a field survey in the vicinity of a riding stable in southwestern France with a reported chronic case of recurrent horse dermatitis to (i) characterize the *Culicoides* species associated with horse populations and (ii) estimate the relative abundance of the different species identified based on the trapping site location.

**Methods:**

For this purpose, three Onderstepoort Veterinary Institute (OVI) traps were set up for one night once a week, from mid-June to the end of July 2022, the known adult peak activity period. Traps were placed either indoors or outdoors at horse facilities.

**Results:**

*Culicoides obsoletus*/*scoticus* were more abundant (58.3%), followed by *C. circumscriptus* (12.1%), *C. nubeculosus* (11.5%), *C. punctatus* (5.6%), *C. festivipennis* (3.8%), *C. pulicaris* (2.3%), *C. riethi* (2.3%), *C. parroti* (2.2%), and the remaining species, *C. lupicaris*, *C. dewulfi*, *C. brunnicans*, *C. flavipulicaris*, and *C. picturatus*, collectively representing only 1.4%. Importantly, *C. obsoletus*/*scoticus* and *C. circumscriptus* were found indoors in notable proportions (54% and 11.4% of captures, respectively).

**Conclusions:**

The findings highlight the continuous exposure of horses to *Culicoides* bites during the warm season, including at night and indoors, from *C. obsoletus*/*scoticus*, but also including low-impact species like *C. punctatus* and *C. pulicaris*. This underlines the need for ongoing research and surveillance.

**Graphical Abstract:**

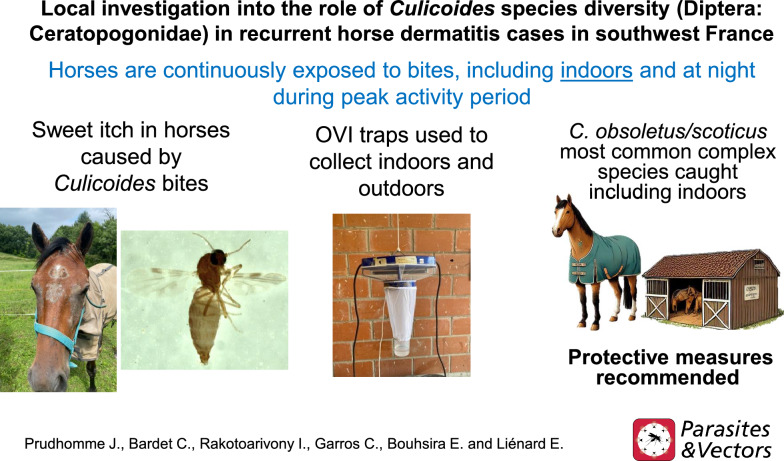

**Supplementary Information:**

The online version contains supplementary material available at 10.1186/s13071-025-06694-2.

## Background

*Culicoides* are small hematophagous biting midges (1–2.5 mm in body size) with scale-free wings and a distinctive light and dark wing pattern, which is used with other morphological features in species identification. In addition to the nuisance to human and animal populations, these insects can impact livestock and human health by transmitting various pathogens. In France, the *Culicoides* species list includes 83 species for mainland France and 61 species for Corsica. Recently, four new species have been recorded in mainland France: *C. manchuriensis* Tokunaga, 1941, *C. abchazicus* Dzhafarov, 1964, *C. ibericus* Dzhafarov, 1963, and *C. saevus* Kieffer, 1922 [1–3].

In mainland France, the most commonly collected species are *C. obsoletus* (Meigen), 1818, and *C. scoticus* Downes and Kettle, 1952 [2]. These two species are usually referred as the Obsoletus complex, because *C. obsoletus* and *C. scoticus* females are morphologically indistinguishable. Other prevalent species include *C. chiopterus* (Meigen), 1830, *C. dewulfi* Goetghebuer, 1936, *C. pulicaris* (Linnaeus), 1758, *C. newsteadi* Austen, 1921, and *C. punctatus* (Meigen), 1804 [2, 4, 5]. Additionally, some species are widely collected in specific regions, such as *C. brunnicans* Edwards, 1939 in western France [6]. In contrast, *C. chiopterus* and *C. dewulfi* are rarely found in Corsica, indicating regional variations in species distribution.

Some biting midge species of the genus *Culicoides* play a crucial role in the transmission of parasitic filariae [7] and act as vectors for several economically significant viruses that affect livestock worldwide, including bluetongue virus (BTV), epizootic hemorrhagic disease virus (EHDV), Schmallenberg virus (SBV), Akabane virus (AKAV), and African horse sickness virus (AHSV) [8–11]. AHSV is closely related to EHDV and BTV, with the latter two viruses currently emerging in Europe. The most recent introduction of AHSV into Europe occurred in Spain in 1987 [12]. The European Food Safety Authority (EFSA) stresses the importance of collecting data on local populations of *Culicoides* to help prepare the best measures in case of a new AHSV introduction [13]. In addition to their impact on livestock, *Culicoides* are vectors for the Oropouche virus, which affects human populations in Central and South America [14].

*Culicoides* are also responsible for causing seasonal allergic dermatitis in horses, commonly known as summer itch or sweet ich. This condition is triggered by allergens present in the saliva of the midges, leading to the most common pruritic dermatitis in horses. In France, 10% of horses are affected by this hypersensitivity [15], which causes severe itching and results in self-inflicted lesions mainly along the dorsal midline [16, 17]. The ventral midline, head, and ears can also be affected [18], leading to secondary infections and impacting the horses' behavior and activity [19]. The first clinical signs of this condition in horses born in Europe are generally observed between 2 and 5 years of age [20].

For the majority of horses, the dermatitis appears in spring, intensifies in summer, and progressively regresses in autumn until it disappears, except in chronic cases where the lesions persist even during winter [17]. This seasonality corresponds to the known peak activity period of *Culicoides* [21]. As this condition is a frequent health problem, we conducted a field survey in a riding school with reported case of recurrent horse dermatitis to (i) characterize the *Culicoides* species associated with horse populations and (ii) estimate the relative abundance of the different species identified during the risk period based on the trapping site location.

## Methods

### Study sites

The study was performed in the South of France, Toulouse region, in the veterinary school equestrian center (43°36′10″N, 1°22′52″E). This region has a transitional oceanic climate, between Mediterranean and continental [22]. The area is characterized by the presence of various potential *Culicoides* hosts, such as domestic animals (e.g., sheep, cows, horses, cats, dogs), as well as many different wild animals (e.g., foxes, rodents, reptiles, birds, amphibians). The 10-ha facility comprises 4 ha of forest, multiple equestrian facilities for training and competitions, and 14 paddocks. Among the 77 horses at the riding center, 16 are stabled at night throughout the year. A third of these horses are club horses, while the others are privately owned. One of the local horses, which arrived at the facility in 2020, a 7-year-old gelding, suffers annually from insect bite hypersensitivity.

Three trapping sites were selected based on their indoor–outdoor location, access to electricity, distance from the adjacent river and forest, and the presence or absence of horses (Table 1).

**Table 1:** Details of the sampling stations in the study area

Station number		A	B	C
Coordinates	North	43°36′08″N	43°36′08″N	43°36′14″N
	East	1°22′52″E	1°22′52″E	1°22′53″E
Altitude (m)		143	142	143
Trap location		Inside	Outside	Outside
Distance (m) from	Stable	0	10	190
	River	143	235	175
	Forest	310	300	125
Number of horses		16	Ø	21

### Specimen collection and identification

Individuals were collected for one night using Onderstepoort Veterinary Institute (OVI) traps, once a week, during June and July 2022. The capture period targeted the *Culicoides* peak of activity [2] which corresponds to the highest risk period for horse dermatitis. In the three sampling sites (Table 1), one OVI trap was set up and operated between 05:30 pm and 08:30 am. Traps were placed at a height of 1.70 m, protected from rain, but exposed to other weather conditions. Each collection tray was filled with 200 ml of soapy water (distilled water mixed with a few drops of liquid dish soap—(Love & Green Ecodetergent, fragrance-free^®^, Love & Green, Rueil-Malmaison, France) before capture. Nets and ultraviolet (UV) lights were checked at each installation and collection. For each trap during capture and collection, data were collected on the following habitat variables: trap position (A, B, or C), presence or absence of horses, number of horses, wind conditions (none, light breeze, medium wind, strong wind), rainfall (none, drizzle, light rain, or heavy rain), and minimum and maximum temperatures (manually recorded using digital thermometers—ama-digit ad 15 th, ± 0.4 °C).

OVI traps are one of the most efficient and sensitive traps for *Culicoides*, capable of capturing even less abundant species [23]. They have been used in several studies and, notably, since 2001 in the *Culicoides* surveillance network in France [1, 24, 25]. Therefore, to facilitate comparison, we used the same capture, sorting, and identification protocols.

Briefly, after each night of capture, dead specimens were collected through a sieve, placed in collection tube, labeled, and stored in 70% alcohol at +4 °C. If the total volume of insects exceeded 3 ml, a subsampling was carried out following previously described protocol [26]. *Culicoides* were identified morphologically according to the determination key of Delécolle [27] and the IIKC database [28]. The following criteria were used: sensory fosse shape and size, number and arrangement of wing spots, spermathecae for females, and aedeagus or parameres for males. Identification was conducted to the species or complex level for morphologically indistinguishable species. Sex and parity were recorded, with parity determined using abdomen pigmentation [9].

### Statistical analyses

Statistical analyses were performed using R studio software [29]. Differences between traps and dates were analyzed with a non-parametric Kruskal–Wallis test, followed by a post hoc analysis using Dunn's test with Bonferroni correction. The effects of the environmental and climatic variables on the *Culicoides* abundance were analyzed using a general linear model (GLM) of analysis of variance (ANOVA) using a quasi-Poisson distribution. The explanatory variables were as follows: presence of rain, temperature (minimum and maximum), presence of horses, and trap position (indoors or outdoors).

## Results

### *Culicoides* fauna

A total of 2181 individuals from 14 species (*C. obsoletus*/*scoticus*, *C. circumscriptus* Kieffer, 1918, *C. nubeculosus* (Meigen), 1830, *C. punctatus*, *C. festivipennis* Kieffer, 1914, *C. pulicaris*, *C. riethi* Kieffer, 1914, *C. parroti* Kieffer, 1922, *C. lupicaris* (as defined by Delécolle, 1983), *C. dewulfi* Goetghebuer, 1936*, C. brunnicans* Edwards, 1939, *C. flavipulicaris* Dzhafarov, 1964, and *C. picturatus* Kremer and Deduit, 1961) were identified in this study. We considered *C. lupicaris* as a distinct species, despite its current acceptance as a synonym of *C. delta*, as evidence indicates that these two are indeed valid and distinct species [30]. The species of the Obsoletus complex, *C. obsoletus* and *C. scoticus*, were the predominant species in the study area (*N* = 1272, 58.3%). Following these, *C. circumscriptus* and *C. nubeculosus* were the next most common species (*N* = 263, 12.1%; and *N* = 250, 11.5%, respectively). *Culicoides punctatus* (*N* = 123, 5.6%), *C. festivipennis* (*N* = 82, 3.8%), *C. pulicaris* (*N* = 51, 2.3%), *C. riethi* (*N* = 50, 2.3%), and *C. parroti* (*N* = 49, 2.2%) were less abundant. The remaining species (*C. lupicaris*, *C. dewulfi*, *C. brunnicans*, *C. flavipulicaris*, and *C. picturatus*) represented only 1.4% of captures (*N* = 17; 9, 2, 2, and 1, respectively) (Tables 2 and 3). A small number of specimens (0.6%) could not be identified due to degradation, and the parity of some females could not be determined (2%). As expected, females (*N* = 2017, 92.5%) were more abundant than males (*N* = 164, 7.5%) across all species and capture sites (Tables 2 and 3). Indeed, light traps are known to preferentially capture adult females [23].

**Table 2:** Number and relative abundance (%) of *Culicoides* species in the study area

Species	Total	Females	Males	Percentage (%)^a^
*C. obsoletus*/*scoticus*	1272	1256	16	58.3
*C. circumscriptus*	263	235	28	12.1
*C. nubeculosus*	250	250	0	11.5
*C. punctatus*	123	106	17	5.6
*C. festivipennis*	82	65	17	3.8
*C. pulicaris*	51	51	0	2.3
*C. riethi*	50	6	44	2.3
*C. parroti*	49	31	18	2.2
*C. lupicaris*	17	13	4	0.8
*C. dewulfi*	9	0	9	0.4
*C. brunnicans*	2	1	1	0.1
*C. flavipulicaris*	2	2	0	0.1
*C. picturatus*	1	1	0	< 0.1
*Culicoides* sp.	10	0	10	0.6
Total	2181	2017	164	100

The details of the captures by traps are summarized in Table 3

^a^Relative abundance: (*n*x/*N*) × 100, where *n*x is the number of individuals belonging to species x, and *N* is the total number of sampled individuals

**Table 3:** Number and relative abundance (%) of *Culicoides* species by trapping site

Trapping site	A				B				C				Overall total
Species	Total	Females	Males	Percentage (%)^a^	Total	Females	Males	Percentage (%)^a^	Total	Females	Males	Percentage (%)^a^	
*C. obsoletus/scoticus*	592	577	15	77.8	138	137	1	45.8	542	542	0	48.4	1272
*C. circumscriptus*	30	30	0	3.9	92	81	11	30.6	141	124	17	12.6	263
*C. nubeculosus*	1	1	0	0.1	1	1	0	0.3	248	248	0	22.2	250
*C. punctatus*	54	54	0	7.1	35	35	0	11.6	34	17	17	3.0	123
*C. festivipennis*	36	36	0	4.7	22	17	5	7.3	24	12	12	2.1	82
*C. pulicaris*	17	17	0	2.2	2	2	0	0.7	32	32	0	2.9	51
*C. riethi*	9	0	9	1.2	6	6	0	2.0	35	0	35	3.1	50
*C. parroti*	1	0	1	0.1	5	5	0	1.7	43	26	17	3.8	49
*C. lupicaris*	0	0	0	0.0	0	0	0	0	17	13	4	1.5	17
*C. dewulfi*	9	0	9	1.2	0	0	0	0	0	0	0	0	9
*C. brunnicans*	0	0	0	0	0	0	0	0	2	1	1	0.2	2
*C. flavipulicaris*	2	2	0	0.3	0	0	0	0	0	0	0	0	2
*C. picturatus*	1	1	0	0.1	0	0	0	0	0	0	0	0	1
*Culicoides* sp.	9	0	9	1.2	0	0	0	0	1	0	1	0.1	10
Total	761	718	43	100	301	284	17	100	1119	1015	104	100	2181

^a^Relative abundance: (*n*x/*N*) * 100, where *n*x is the number of individuals belonging to species x, and *N* is the total number of sampled individuals

### Influence of trapping sites and climatic conditions

Thunderstorms (June 24 and 30) and heavy rains (July 23) likely reduced insect activity or trap efficiency, negatively impacting captures at the three trapping sites. Additionally, a malfunction of the black light neon prevented captures in trap A on June 30.

For the number of *Culicoides* captured, all species combined, differences between traps were not significant (Kruskal–Wallis test, *P* = 0.054). Similarly, no significant differences were observed for *C. brunnicans*, *C. circumscriptus*, *C. dewulfi*, *C. festivipennis*, *C. flavipulicaris*, *C. obsoletus/scoticus*, *C. parroti*, *C. picturatus*, *C. pulicaris*, *C. punctatus*, and *C. riethi* (Kruskal–Wallis test, all *P* > 0.05) between traps. However, two species *C. lupicaris* (Dunn’s test, *P* = 0.01) and *C. nubeculosus* (Dunn’s test, *P* = 0.013) were captured in higher abundance in trap C than in traps A and B. All the species present at capture site B were also found in capture site A. Nevertheless, *C. flavipulicaris* (*N* = 2), *C. picturatus* (*N* = 1), and *C. dewulfi* (*N* = 9) were only recorded in trap A. The number of *Culicoides* captured did not significantly differ by capture date throughout the risk period for any species (Kruskal–Wallis test, all *P* > 0.01).

Regarding the impact of climatic conditions, analyses were not performed for *C. lupicaris*, *C. dewulfi*, *C. brunnicans*, *C. flavipulicaris*, and *C. picturatus* due to the low number of individuals captured (Table 3). Analysis using ANOVA demonstrated that meteorological and environmental factors significantly influenced the activity of three species: *C. circumscriptus*, *C. nubeculosus* and *C. parroti* (Table 4). These species were mainly captured in trap C, which was located outside near the paddocks and the river. Furthermore, individuals were captured in trap A (indoor trap) when there was a medium-speed wind (average of 15 km/h, Beaufort scale 3/12), but a decrease in captures was observed in each trap when winds were strong (average > 20 km/h, Beaufort scale 4/12).

**Table 4:** Detailed *P*-values obtained

Variables	Rain	T max	T min	Horse presence	Trap position
*C. obsoletus/scoticus*	0.979	0.133	0.928	0.06	0.881
*C. circumscriptus*	< 0.001 ***	< 0.001 ***	0.156	0.811	< 0.001 ***
*C. nubeculosus*	0.707	< 0.01 **	< 0.01 **	< 0.001 ***	< 0.001 ***
*C. punctatus*	0.224	< 0.05 *	0.135	0.676	0.442
*C. festivipennis*	0.094	0.09	0.893	0.672	0.605
*C. pulicaris*	0.134	0.925	0.337	< 0.05 *	0.414
*C. riethi*	0.265	0.81	0.171	0.322	0.251
*C. parroti*	< 0.05 *	< 0.01 **	0.868	0.055	< 0.001 ***

*P*-value significance: ***0.01, **0.01, *0.05

### Parity rate

The low parity rate (53.5%) reflects a relatively young population. We observed a peak of nulliparity on July 8, indicating an emergence of *Culicoides*. Breeding sites were likely located close to the traps (horse manure on the farm, for example). No significant differences in parity were found between traps (*P* = 0.19) or between dates (*P* = 0.11).

## Discussion

Recurrent horse dermatitis remains a significant health concern affecting a considerable number of horses, around 10% of the world's equine population [31]. This study focuses on the *Culicoides* species that may contribute to this condition in southwest France, emphasizing the potential risk to susceptible horses from exposure to various *Culicoides* species. Identified species, such as *C. obsoletus*/*scoticus* [32], *C. pulicaris* [33], *C. nubeculosus* [16], *C. lupicaris* [34], *C. punctatus* [16], and *C. circumscriptus* [35], are known or suspected causes for this condition. This study also offers key recommendations to better protect horses from these insects.

### *Culicoides* fauna

A mere 7.5% of *Culicoides* males were captured, consistent with prior studies [25, 36]. OVI traps, being attractive to nocturnal females seeking blood meals, also did not capture diurnal *Culicoides*. The observed wave of nulliparity on July 8 suggests the proximity of larval sites, as adult dispersal is limited, with most individuals found near breeding sites [37]. Recent works have shown that abdomen pigmentation is not an accurate indicator of parity and could be biased by dietary components associated with organically enriched substrates [38]. In Europe, *Culicoides* larvae are found in a variety of habitats, which can range from muddy areas on farms rich in organic matter to more undisturbed environments. While some larvae may occur in areas trampled by animals, most habitats tend to be relatively undisturbed, as *Culicoides* are not strong swimmers and drown easily [9, 39]. During the study period, few species' abundances were influenced by climatic and environmental fluctuations, except for the negative impacts of strong winds and heavy rainfall, as commonly observed [40].

Species like *C. obsoletus* and *C. scoticus* are predominant in France, particularly in temperate zones [21, 41]. These species exhibit a wide range of mammalian hosts and thrive in the favorable climatic conditions of the region. Although their abundance was not significantly influenced by the presence of horses in our study, other research has shown their tendency to enter livestock buildings, especially in the presence of animals or during unfavorable weather conditions [4, 42].

In contrast to the national *Culicoides* surveillance network's findings of low abundance [2], *C. circumscriptus*, *C. nubeculosus*, *C. festivipennis*, and *C. punctatus* showed higher abundance levels in our study. These variations could be explained by differences in nearby hosts, as the surveillance network primarily targeted ruminant farms. Notably, *C. circumscriptus* and *C. festivipennis* are known to feed frequently on birds [43]. These species were particularly abundant in the outdoor trap near the forest, possibly due to the presence of bird nests in the vicinity. *Culicoides nubeculosus*, accounting for 11.5% of total captures, emerged as the fourth most frequent species. Surprisingly, only one individual of *C. nubeculosus* was captured inside during our study. This contrasts with its known preference for horses [44] and its higher abundance in other countries [45]. The discrepancy could be attributed to favorable climatic conditions and behavioral responses to weather. This species is also a potential vector for *Onchocerca cervicalis* [46]. Given the similarities between *Onchocerca* infections and summer itch symptoms, and the small number of studies investigating the prevalence in vectors, further research on parasite prevalence should be added in future studies.

Although commonly the second most frequent species around horses in the Netherlands, *C. pulicaris* ranked seventh in our study, aligning with previous estimates of around 5% of captures [47]. In the United Kingdom, *C. pulicaris* has been directly linked with insect bite hypersensitivity in horses [33]. Therefore, despite its lower abundance, *C. pulicaris* remains a species of concern due to its feeding preferences (horses and farm animals) and activity period in the southeast of France from April to September [3].

*Culicoides brunnicans* and *C. dewulfi* were captured in low numbers (two and nine individuals, respectively). This low capture rate may be attributed to their distinct ecological and behavioral traits. *Culicoides brunnicans* is primarily exophagic [42], meaning it feeds outdoors and is less likely to be attracted to traps positioned near or inside buildings. Conversely, while *C. dewulfi* is endophagic [42] and prefers indoor feeding, its relatively low abundance in our study might reflect its habitat preferences, specific host associations, or lower local population density at the trapping sites.

Finally, *C. newsteadi*, observed abundantly by the national surveillance network (10% of the capture) [2], was not collected during our study. This discrepancy could be attributed to differences in feeding preferences and habitat preferences, with *C. newsteadi* primarily feeding on cattle, sheep, and humans, and being more prevalent in coastal areas at lower altitudes [43, 48].

### Risk and recommendations

Our study underscores the potential exposure of horses to various *Culicoides* species, indicating the risk of sweet itch in susceptible horses. Among the identified species, *C. obsoletus*/*scoticus*, *C. pulicaris, C. nubeculosus*, *C. lupicaris*, *C. punctatus*, and *C. circumscriptus* are known to be responsible for insect bite hypersensitivity in horses [16, 32, 35, 47, 49, 50]. However, the impact of species like *C. punctatus*, *C. pulicaris*, and *C. lupicaris* might be minor, given their low capture percentages (5.6%, 2.3%, and 0.8%, respectively).

Current guidance highlights several effective strategies, including the application of pyrethroids to livestock and their housing, the use of midge-proof stables for high-value or viremic animals, and the adoption of proper farm management practices to help minimize local breeding habitats [33]. In France, a commonly recommended non-chemical approach to protecting horses involves stabling them from late afternoon until the following morning [47, 51]. While this may be effective for species like *C. nubeculosus*, predominantly found outdoors, it might not be sufficient for species like *C. obsoletus*/*scoticus* and *C. circumscriptus*, which were found in large quantities indoors as previously reported in cattle farms [52]. Additionally, as temperatures decrease, an increase in the number of individuals captured indoors was observed. Therefore, effective implementation of this recommendation requires ensuring no open doors or windows and using tightly fitted mosquito nets [53, 54]. Given horses' susceptibility to heat stress [55], installing light traps and vertical high-speed fans opposite the entrances in stables could be considered [54]. Moreover, horses affected by sweet itch could be covered with anti-insect blankets impregnated with repellents like deltamethrin or permethrin. To avoid the creation of breeding sites for *Culicoides*, it is necessary to ensure general hygiene. Ideally, manure should be removed twice a day from the stalls and paddocks. Stagnant water, leaking irrigation pipes, overflowing troughs, and wet areas should be eliminated.

## Conclusions

Our study highlights the potential risk of sweet itch in susceptible horses due to exposure to various *Culicoides* species. Identified species such as *C. obsoletus*/*scoticus*, *C. pulicaris*, *C. nubeculosus*, *C. lupicaris*, *C. punctatus*, and *C. circumscriptus* are known to be common causes of insect bite hypersensitivity. However, the impact of species like *C. punctatus*, *C. pulicaris*, and *C. lupicaris* may be minor, given their low capture percentages. Our findings emphasize the importance of implementing protective measures such as stabling horses and using anti-insect blankets, particularly against prevalent indoor species. Effective control strategies require continuous surveillance and further research to better manage this persistent health concern affecting horses.

## Supplementary Information

Additional file 1: Table S1. Raw data on *Culicoides* species: temporal and species-specific capture records.

## Data Availability

The authors confirm that all data underlying the findings are fully available without restriction. All relevant data are within the paper and its Supplementary Information files.
